# Supplementary material for: Pangenomic analysis reveals plant NAD+ manipulation as an important virulence activity of bacterial pathogen effectors
Source: Proc Natl Acad Sci U S A. 2023 Feb 8;120(7):e2217114120. doi: 10.1073/pnas.2217114120 (PMC9963460; doi:10.1073/pnas.2217114120)
Supplement: Supplementary file 1 — Appendix 01 (PDF) [file pnas.2217114120.sapp.pdf]

PNAS

Supporting Information for

Pangenomic analysis reveals plant NAD$^+$ manipulation as an important virulence activity of bacterial pathogen effectors

Michelle T. Hulin[1], Lionel Hill[2], Jonathan D. G. Jones[1], Wenbo Ma[1*]

[1] The Sainsbury Laboratory, Norwich Research Park, NR4 7UH, United Kingdom

[2] John Innes Centre, Norwich Research Park, NR4 7UH, United Kingdom

[*] Corresponding author: wenbo.ma@tsl.ac.uk

This PDF file includes:

- Supplemental methods
- Tables S1-5
- Legends for Datasets 1-3
- Figures S1-S10
- SI references

Other supporting materials for this manuscript include the following:

- Dataset S1-S3

Supplementary methods

Bacterial strains and plant material

Strains and plasmids used in this study are listed in Table S3. *Pseudomonas* strains used in this study include *P. syringae* pv. tomato strain DC3000 (PstDC3000), the T3S mutant Δ*hrcC* (1), and the effector-less mutant D36E (2). *P. coronafaciens* NCPPB 2397 (equivalent to ICMP 4457, genome assembly GCA_001400695) was used to clone the novel T3E OG18056. Antibiotics were used at the following concentrations kanamycin: 50 µg/mL, rifampicin: 50 µg/mL, carbenicillin: 100 µg/mL, gentamycin 50 µg/mL. *Agrobacterium tumefaciens* strain GV3101 was used for *Agrobacterium*-mediated transient expression in *N. benthamiana*.

N. benthamiana plants were grown in a controlled growth room with 16:8 light:dark conditions at 22 °C, 80% humidity. *A. thaliana* col-0 and *eds1-2* mutant plants were grown in a growth chamber with 10:14 light:dark conditions at 20 °C, 80% humidity. Five week old *N. benthamiana* and four week old *A. thaliana* were used for agro-infiltration and *P. syringae* inoculations.

Plasmid construction

Golden gate cloning (3) was used to generate constructs for transient expression of effectors in plants. Level 0 parts included: 35S long promoter, CDSNS (no stop) acceptor, 3xFLAG C-terminal tag, 35S terminator. The CDS were ordered from Twist Bioscience and PCR amplified using Phusion HF polymerase (ThermoFisher) to add golden gate compatible overhangs. Primers used in this study are presented in Table S4. CDS were inserted into the level 0 CDSNS acceptor using one-pot digestion-ligation reaction with the restriction enzyme BpiI-HF (New England Biolabs). Level 0 fragments were then assembled into the *in planta* expression vector pICH47732.

The *OG18056* effector gene was cloned into pUCP20TK for expression in *P. syringae*. Genomic DNA containing CDS and its native promoter (250 bp upstream of the start codon) was amplified from *P. coronafaciens* NCPPB 2397 with the addition of a C-terminal HA tag in the reverse primer. For *hopAM1*, the wild-type allele (encoded by PstDC3000) and E191A mutant were synthesized with C-terminal HA tags (Twist Bioscience) and PCR amplified to add overhangs for infusion cloning (Takara).

The *hopAM1* promoter sequence (207 bp upstream of the start codon) was PCR amplified from PstDC3000 gDNA. Infusion cloning was used to insert promoter and CDS fragment(s) in reverse orientation to the *lacZ* promoter in the pUCP20TK vector after linearisation of the vector with BamH1 and EcoR1 (New England Biolabs). For generation of the E305A mutant of OG18056, a site-directed mutagenesis approach using infusion was performed, splitting the gene and promoter fragment into two PCR products for cloning with the mutation introduced in primers R1 and F2. All constructs were confirmed by sanger sequencing. The recombinant plasmids were electroporated into *P. syringae* as described in (4) apart from recovery growth was for 2 hours at 28 °C, 220 rpm. The vectors pBBR1MCS-5 and pBBR1MCS-5::avrRps4 (5) were also introduced in *P. syringae* D36E via electroporation.

Agro-infiltration in *N. benthamiana*

A. tumefaciens GV3101 with various constructs was streaked from glycerol stocks onto LBA plates containing appropriate antibiotics. Two-day old bacterial lawns were resuspended in sterile distilled water and washed twice by spinning at 3000 rpm for 10 minutes. The cells were resuspended in $MgCl_2$-2-(N-morpholino)ethanesulfonic acid (MES) buffer (10 mM $MgCl_2$, 10 mM MES, pH 5.6) to a final concentration of OD_{600}=1.0 before adding acetosyringone to a final concentration of 150 μM. Bacteria were incubated at room temperature (22 °C) for 3 hours before combining in a 1:1 ratio with the strain carrying the viral RNA silencing suppressor P19, meaning the final OD_{600} of each *Agrobacterium* strain was 0.5. Protein expression was confirmed by immunoblotting using total proteins extracted from 1 cm leaf discs at 2 days post inoculation. Leaf discs were immediately frozen in liquid nitrogen and stored at -80 °C. To extract proteins, 100 µL of protein extraction buffer (50 mM Tris-HCl (pH 7.5), 150 mM NaCl, 1 mM EDTA, 0.1% Triton X-100, 10mM DTT, 1x cOmplete EDTA-free protease inhibitor cocktail (ThermoFisher)) was added and the discs were ground with a micropestle whilst on ice. Crude plant material was removed by centrifugation at 11,000 x g for 15 minutes and 60 µL of the supernatant was combined with 30 µL 3X Laemmli buffer (6) and boiled at 95 °C for 5 minutes. Immunoblotting was performed as described previously (7). An anti-FLAG antibody conjugated to horseradish peroxidase (Sigma) was used at 1:5000.

Immunoprecipitation of proteins was performed using 0.5 g of leaf material ground in liquid nitrogen and then 1 mL of protein extraction buffer (as above) added before centrifugation at 11,000 x g for 15 minutes. Input samples before addition of beads were included for comparison. 10 µL/mL of anti-FLAG M2 affinity Gel (A2220 Sigma-Aldrich) was added to cleared samples and incubated at 4°C with gentle agitation for 2 hours. Beads were washed four times for 5 minutes in protein extraction buffer before resuspension in 60 µL protein extraction buffer. Immuno-precipitated proteins were visualised by immunoblot as above by boiling 20 µL of beads in Laemmli buffer.

Effector expression and secretion analysis

To confirm that novel effector protein OG18056 was induced in Hrp-inducing minimal medium and secreted in a T3S-dependent manner, PstDC3000 and the ∆*hrcC* mutant carrying pUCP20TK::OG18056-HA were used for protein extraction. A protocol based on (8) was utilised to extract secreted proteins from supernatants. The protocol was modified to extract from 8 mL of bacterial supernatant (rather than 2 mL). Immunoblotting was performed as in (7) using anti-HA from rat (Roche) at 1:1000, followed by a secondary anti-rat antibody conjugated to horseradish peroxidase (sc-2750, Santa Cruz) at 1:2000. The same protocol without supernatant extractions was used to confirm the expression of OG18056 and HopAM1 in PstDC3000 D36E used in bacterial infection assays.

***In planta Pseudomonas* pathogenicity assays**

PstDC3000 D36E carrying specific effector alleles or the pUCP20TK empty vector were grown from glycerol stock for 2 days on agar plates at 28 °C, and the lawn was resuspended in 10 mM $MgCl_2$ to the required optical density for inoculation. 1 x 10^8 cfu/mL (OD 0.2) or 5 x 10^6 cfu/mL (OD 0.01) was used to inoculate *A. thaliana* and monitor symptom development over several days, with pictures taken 1 or 3 days post inoculation. For bacterial population assays, inoculum was prepared at 5 x 10^5 cfu/mL (OD 0.001) for *A. thaliana*. Bacteria were enumerated after 0 and 3 days by plating a dilution series on Kings B agar supplemented with rifampicin and kanamycin/gentamycin. All experiments were performed three times.

NADase assay

Semi-*in vitro* NADase assays were performed as in (9) with some modifications. After immuno-precipitation of proteins, 20 μL protein-laden FLAG beads were incubated with 6 μL 100 μM NAD^+ (Merck), 3.84 μL 10x PBS, 1 μL 0.6M NaCl, 1 μL 0.6M $MgSO_4$ and 28.16 μL water (total of 60 μL). The reactions were terminated after 1 hour at 28°C with shaking at 1000 rpm by pulling the beads to one side with a magnet and the supernatant was used directly for LCMS analysis. This experiment was repeated three times with similar results. The AbTIR 2'cADPR standard was extracted from induced *E. coli* lysate as in (10).

Metabolite extraction *Arabidopsis thaliana* and LCMS analysis

P. syringae D36E expressing effectors or just carrying the empty vector were inoculated into *A. thaliana* Col-0 leaves at OD 0.5 (~ 2.5 x 10^8 CFU/mL) and harvested 18 hpi. Each sample consisted of 8 leaves harvested from two plants. Samples were freeze-dried for 48 hours. 0.01g of freeze-dried material was then extracted using 10% methanol 0.1% acetic acid as in (11). This experiment was repeated three times with similar results. NAD^+ depletion assays were performed as above but instead taking samples at day 0 and day 3 and the bacteria were inoculated at a lower dose (~ 5 x 10^6 CFU/mL).

To visualise LCMS peaks Agilent software MassHunter Qualitative was utilised. The EIC of 542.06-542.07 m/z was extracted and data plotted for cADPR. For NAD^+ the EIC of 664.11-664.12 m/z was extracted. For quantitative analysis of NAD^+ the MassHunter Quantification software TOF (Quant-My-Way) was used. NAD^+ was set as the compound with a m/z of 664.11 with retention time of 4.2 minutes with allowance of 0.1 minutes either side. A calibration curve was generated with NAD^+ dilution series from 0.078 μM, 0.3125 μM, 1.25 μM, 5 μM, 10 μM, 15 μM, 20 μM NAD^+. Peaks were gaussian smoothed and areas were integrated automatically and then manually inspected and adjusted if necessary.

Genome retrieval and annotation

2,437 *P. syringae* (taxid136849) genomes were download from NCBI on November 18th, 2020. Genomes were grouped into 806 clusters showing ≥ 99.95% ANI using

FastANI v1.32 (12). One genome per cluster was chosen based on the assembly with the lowest number of contigs. Genomes were filtered using an approach similar to Levy*, et al.* (13), removing those with ≥ 1000 contigs and an N50 value lower than 40,000 bp computed with Quast (14). Finally, CheckM (15) was used to select genomes ≥ 95% complete and < 5% contamination, leaving 531 genomes. Using a cut-off of ≥ 80% ANI, all but two genomes are clustered into a single group. These two genomes (GCA_ 900582625 and GCA_900573885) were kept as out-groups.

Genome annotation was performed using PROKKA (16) and Orthofinder (17) was used to define orthogroups. To generate a core genome phylogeny, 409 single copy orthogroups present in all genomes were aligned using ClustalW (18) with ends trimmed by Gblocks (19). Sequences were then concatenated into a 68,325 bp alignment, converted to the phylip format and input into IQ-TREE2 (20) with parameters JTT+I+G and 1000 bootstraps.

Identification of Type III secretion system (T3S) and known Type III effectors (T3Es)

To identify genomes containing a canonical *hrp* T3S, a BLAST-based approach was taken similar to that described in Dillon et al. (2019). T3S proteins from PstDC3000, *P. syringae* pv. *syringae* B728A, and *P. viridiflava* LP23.1a (Table S5 lists accession IDs) were used in a tBLASTn to query each genome. A genome was determined to putatively encode a *hrp* T3S if it had a hit to every query with > 30% length and > 30% identity for at least one of the three T3S inputs. A similar approach was taken to determine which orthogroups corresponded to known T3E families in *P. syringae*. A set of T3E protein sequences from pseudomonas-syringae.org was used as input to query all orthogroups with BLASTP. Orthogroups containing proteins with hit identities of 100% and E-value < $1x10^{-10}$ were determined to be those corresponding to known T3Es.

Identification of homologues of OG18056

The sequence of OG18056 from *P. coronafaciens* was searched against the non-redundant protein database on NCBI in August 2022, using BLASTP. All hits were extracted and full-length proteins were aligned using the structural-aware alignment program PROMALS-3D (21). The alignment was then used to create a phylogenetic

tree using IQ-TREE (20) with model WAG+I+G4. To compare genomic regions, all genomes with a hit on BLASTP were downloaded and the genomic region was identified manually using Geneious Prime (2022.0.1). Upstream hrp (GGAACC(15-17N)CCACNNA) or PIP (TTCGC(15N)TTCGC) promoter motifs were identified as well as neighbouring gene ontologies. To identify if each genome possessed particular type 3 secretion systems, SctV homologues from five distinct T3SS (Q5K5P1_ECOLX, HRCV_RALSO, INVA_SALTY, Q9I327_PSEAE, Y4YR_SINFN, Q887B4_PSESM) were searched against each genome using tBLASTn. Present T3SS's were determined by assessing the best hits to each SctV homologue. To assess T6 effectors, all OG18056 homologues were uploaded to the SecReT6 online search engine (22) which predicts T6 effectors. In addition, *hopBY* homologues that were within T6 machinery gene clusters on the genome were also considered as possible T6 effectors.

Protein structure prediction and alignment

Structural prediction was conducted using ColabFold AlphaFold2 batch with MMseqs2 (23). Structural alignments were performed with fr-tm-align (24). This included alignments with hSARM1 (structure 6O0R) residues 561-700 aa and hCD38 (structure 6VUA). Aligned residues, root-mean-square deviation (RMSD) scores, and alignment lengths were extracted using bash scripting.

Molecular docking of NAD^+

Autodock Vina v1.2.3 (25) was used for molecular docking. P2Rank (26) was first used to predict catalytic pockets. Output files were processed with python to extract the pocket containing the catalytic residues that aligned with hSARM1-TIR. Pocket coordinates were added to the Vina config file for the docking run. The NAD^+ ligand was downloaded in 3D format from https://zinc20.docking.org (27) and converted to PDBQT format using the Autodock Vina prepare_ligand script. The Radius of Gyration was calculated and multiplied by 2.9 using python to generate the appropriate sized grid for ligand binding (28). Autodock Vina was then ran with exhaustiveness of 32 to produce eight possible models.

Figure creation

PyMOL (https://pymol.org/2/) was used for visualisation of protein structures. WebLogo (http://weblogo.threeplusone.com/) was used to generate a sequence logo of residues at particular positions in OG18056 homologs. Biorender (BioRender.com) was used to create diagrams. All other figures were created using R software (29). Specifically, the packages ape (30), phangorn (31), gggenes (32), ggplot2 (33), ggtree (34) and reshape (35).

Statistical analysis

Analysis of variance (ANOVA) was used to statistically compare population counts of *P. syringae* from *Arabidopsis*. Tukey-HSD significance groups were extracted using R packages emmeans (36) and the cld function within agricolae (37) .

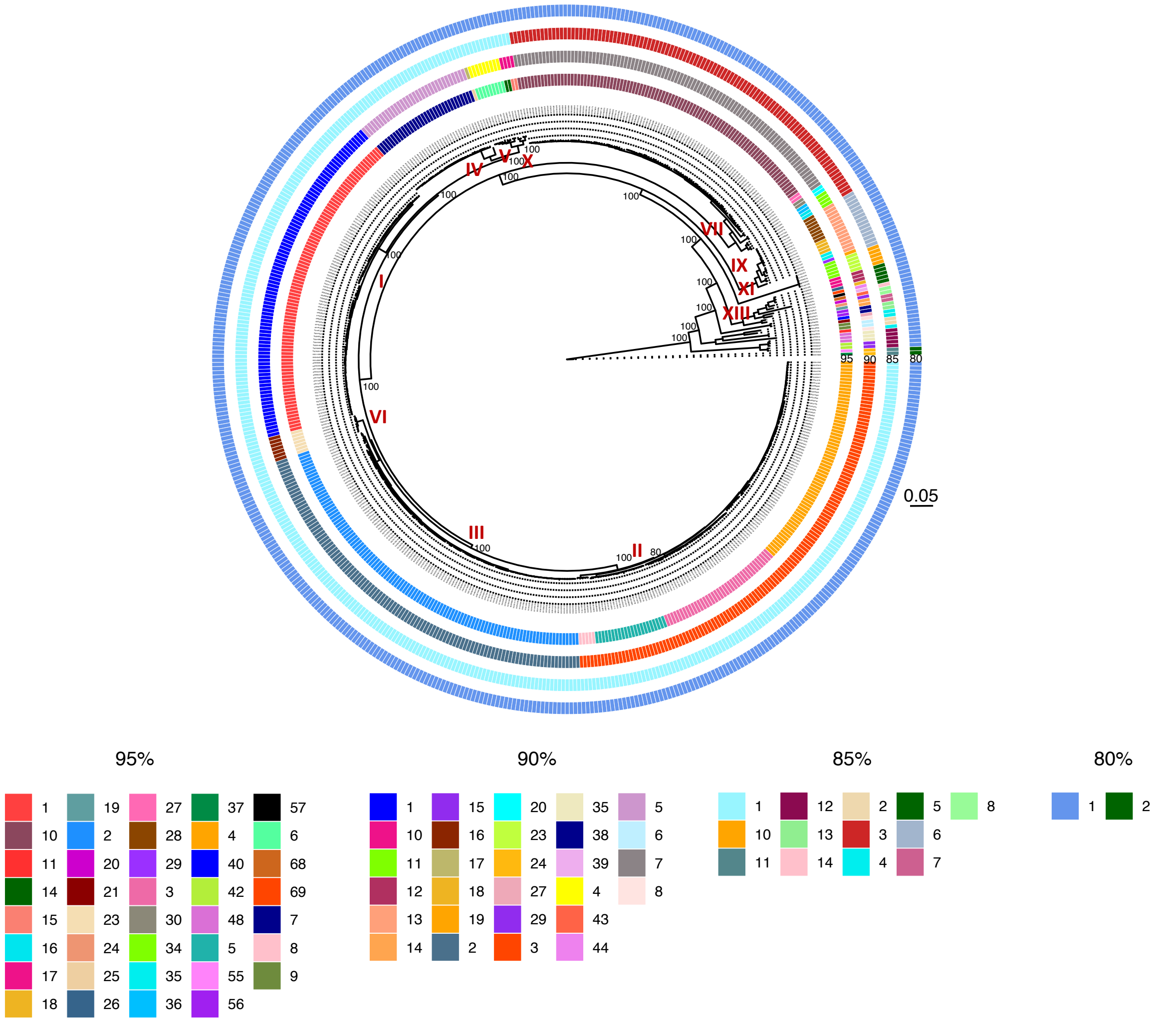

Figure S1. Grouping *Pseudomonas syringae* by core genome and Average Nucleotide Identity (ANI).

The maximum-likelihood core genome phylogeny was based on 409 single copy proteins encoded in all 531 genomes. Circles around the edge group genomes by Average Nucleotide Identity (ANI) calculated using FastANI at the 95%, 90%, 85% and 80% identity level. The corresponding groups are color-coded. Previously defined phylogroups are annotated onto the tree. The tree was rooted using *Pseudomonas* strains GCA_ 900582625 and GCA_900573885 that are divergent from *P. syringae*. The scale shows substitutions per site.

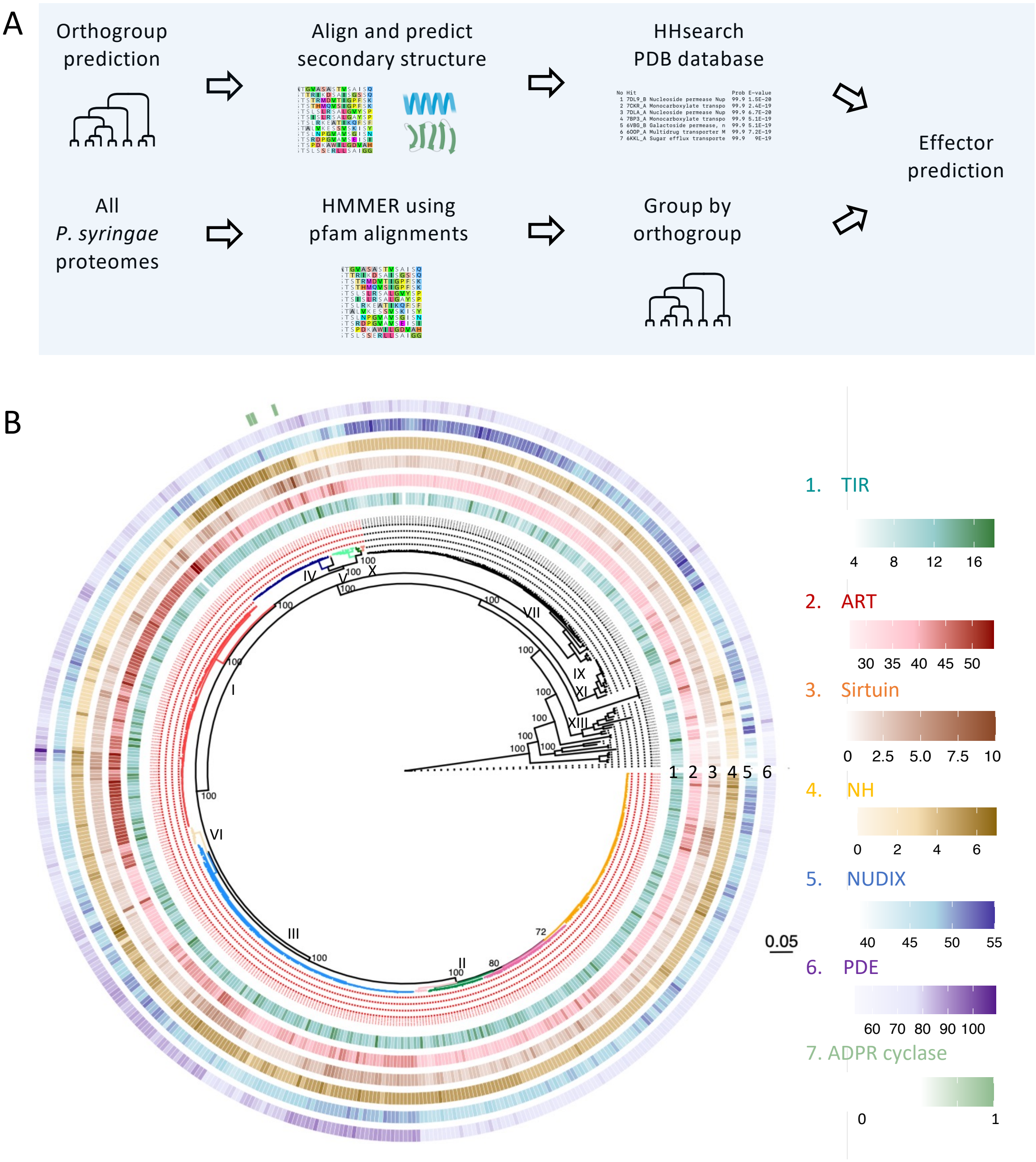

Figure S2. Identification of NADases encoded in the *P. syringae* species complex.

A. Bioinformatic pipeline illustrating the prediction of NAD^+ hydrolyzing enzyme families using HMMER and HHsearch in the *P. syringae* species complex.

B. Presence of NADases predicted across the *P. syringae* species complex is mapped onto the maximum-likelihood core genome phylogeny. Numbers of predicted proteins from each enzyme family is presented by heatmaps. Strains in the primary phylogroups, which mostly rely on the Hrp1 T3S for pathogenicity, are colored in red.

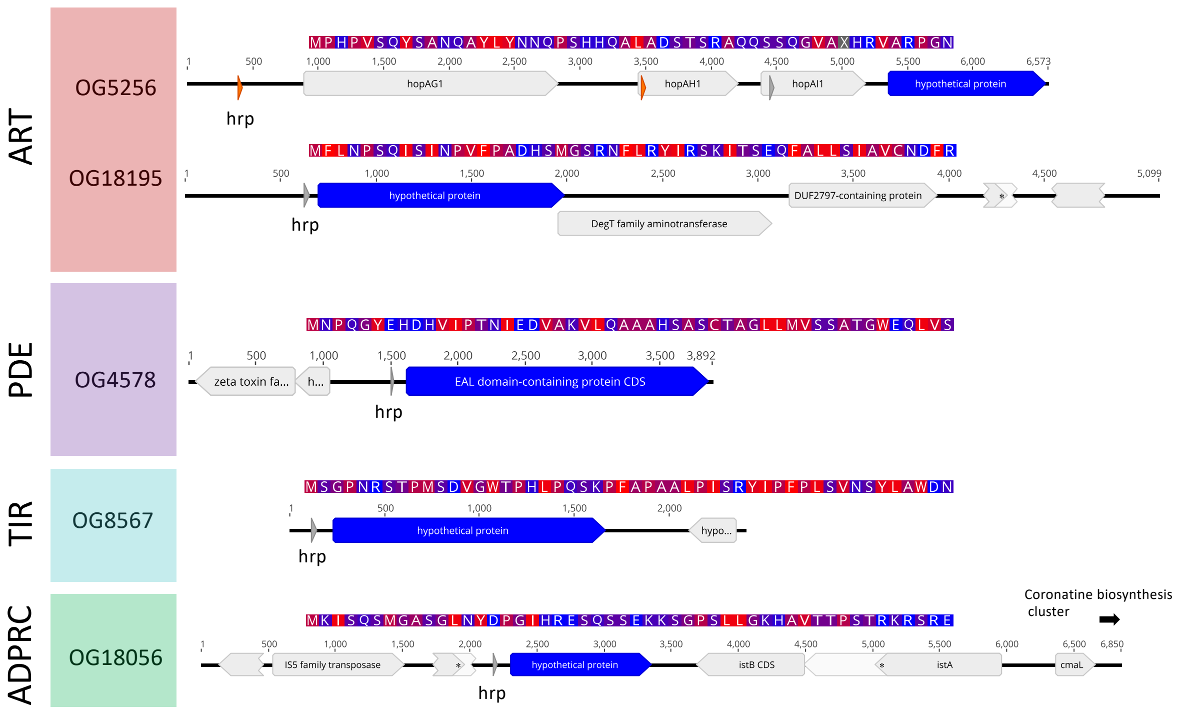

Figure S3. N-terminal sequence predicted to be secretion signal (above) and the genomic location showing the predicted *hrp* box (below) of genes encoding the newly identified type III effector candidates.

The type III-dependent secretion signal is predicted using the consensus amino acid sequence from a multiple sequence alignment of each effector candidate. The genomic location is from a representative genome. The location of *hrp* box in the promoter sequence is highlighted with a grey box. For OG5256, two possible alternative upstream *hrp* boxes (colored in orange) are shown as this gene may be part of an effector operon.

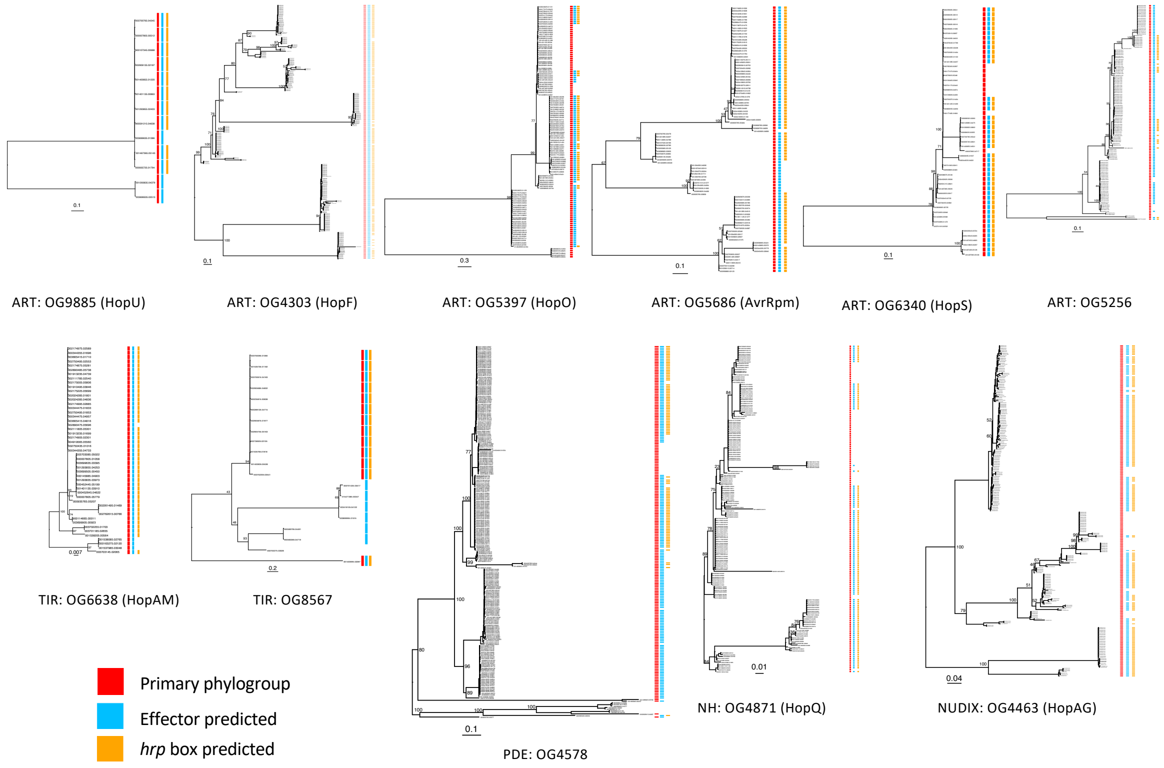

Figure S4. Phylogenetic trees of NADase T3Es in *P. syringae*.

Maximum-likelihood phylogenies were generated using IQ-TREE for all proteins in each orthogroup with < 50% gaps (to remove truncated proteins). The colored bars beside each tree label each sequence for: 1) whether it is from a primary phylogroup genome (red); 2) whether it was predicted as an effector (blue); and 3) whether it has a predicted *hrp* box in its promoter (orange).

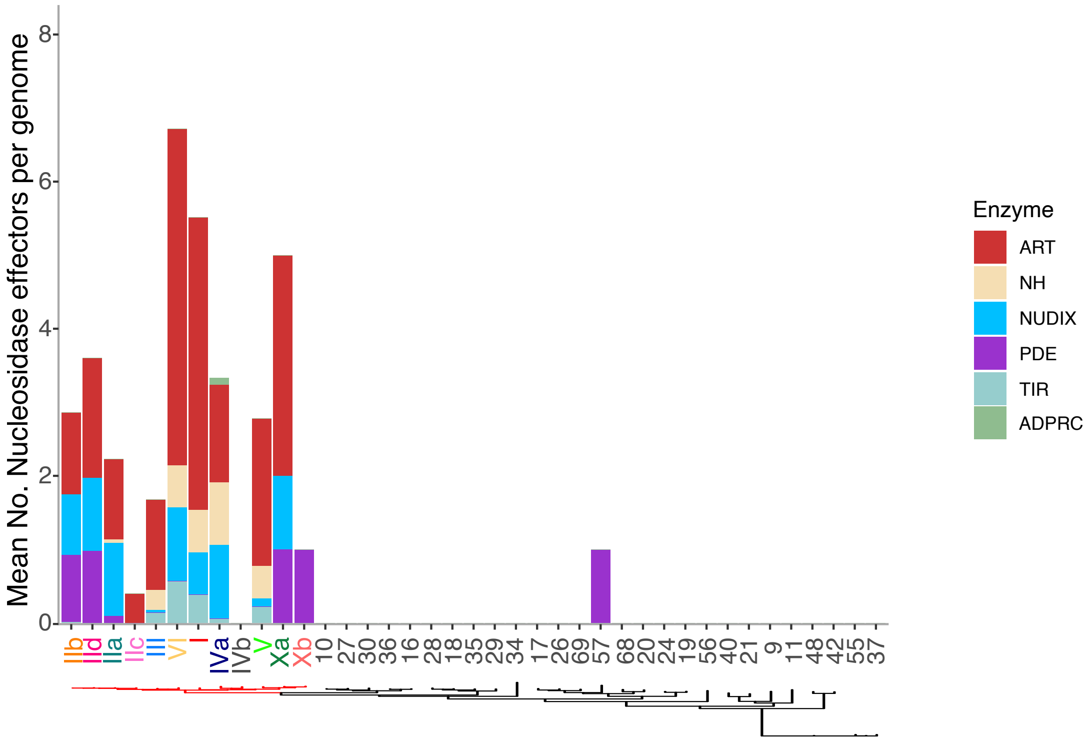

Figure S5. Number of NADase T3Es encoded in each phylogroup of *P. syringae* species complex.

The primary phylogroups are labelled with roman numerals. The Arabic numbers represent secondary phylogroups corresponding to their group number in Fig S1. The mean number of predicted NADase T3Es in each enzymatic family are shown in a collapsed version of the core genome phylogeny with the primary phylogroup clade branches labeled in red. ART: ADP-ribosyltransferase, NH: Nucleoside hydrolase, PDE: phosphodiesterase, ADPRC: ADP-ribose cyclase

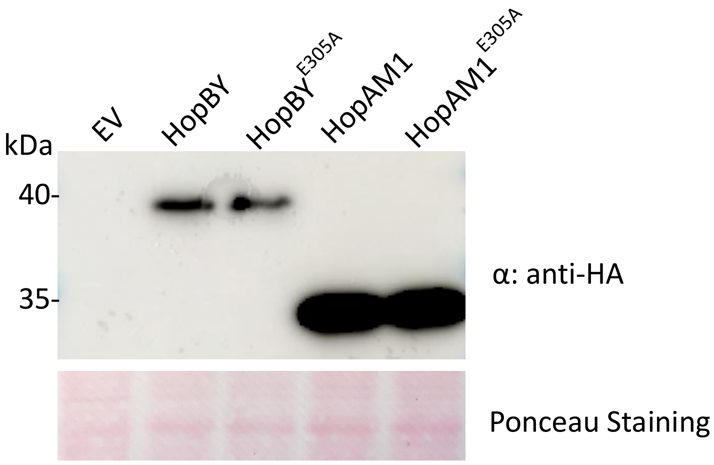

Figure S6. Immunoblots showing the T3Es and their catalytic mutants were expressed in *P. syringae* mutant D36E.

HopBY and HopAM1 were tagged with C-terminal HA. The genes were under their native promoters and cloned in the vector pUCP20TK. D36E carrying the recombinant plasmids were grown in Hrp-inducing minimal medium for 20 hours before cells were collected for western blotting using anti-HA antibody.

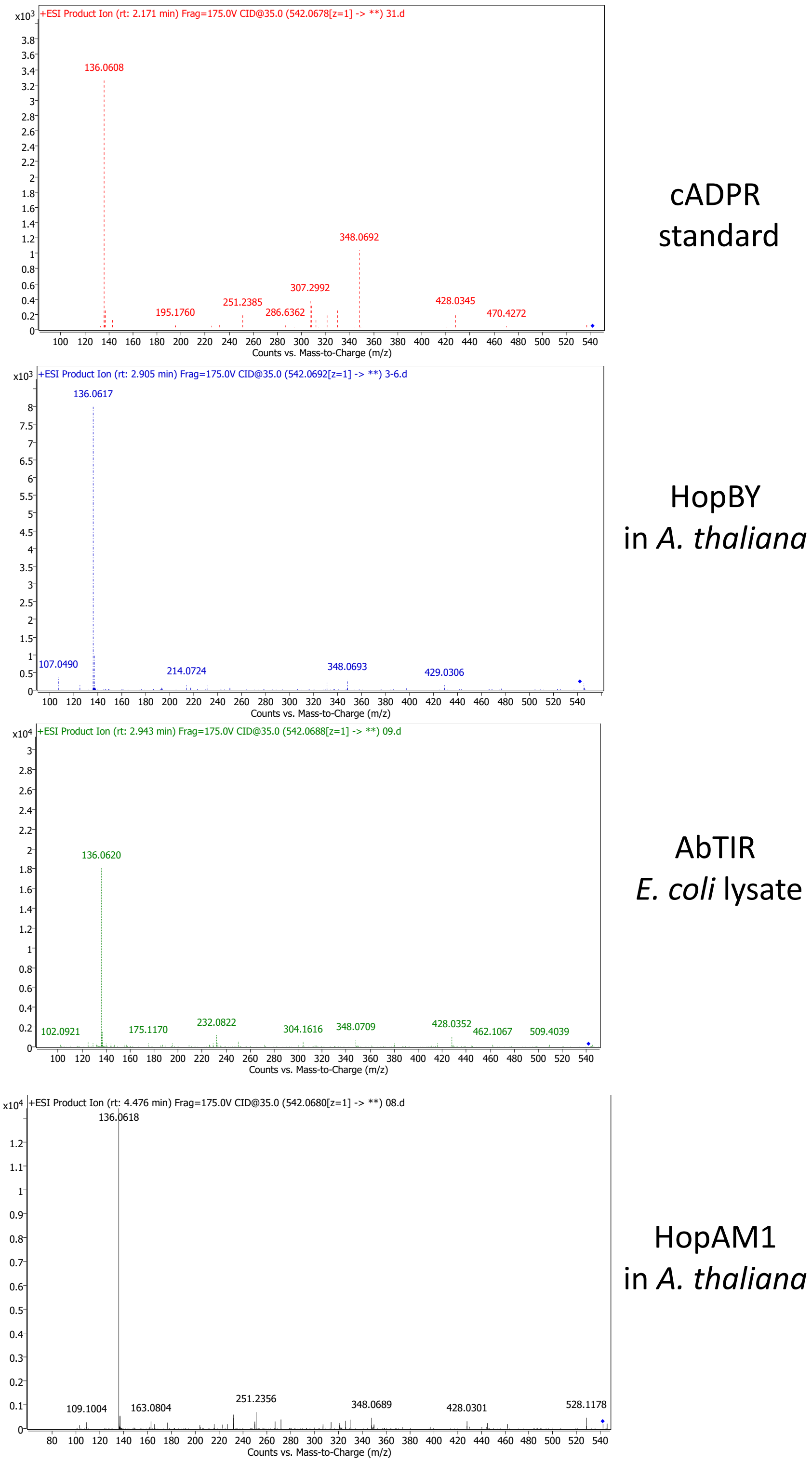

Figure S7. MS/MS spectra of product ion scans of *m/z* 542.06-542.07.

The samples include the cADPR standard, HopBY (*A. thaliana* leaf tissue inoculated with *P. syringae* D36E expressing HopBY), AbTIR (*E. coli* lysate) and HopAM1 (*A. thaliana* leaf tissue inoculated with *P. syringae* D36E expressing HopAM1). The blue diamonds in the spectra represent *m/z* 542.06.

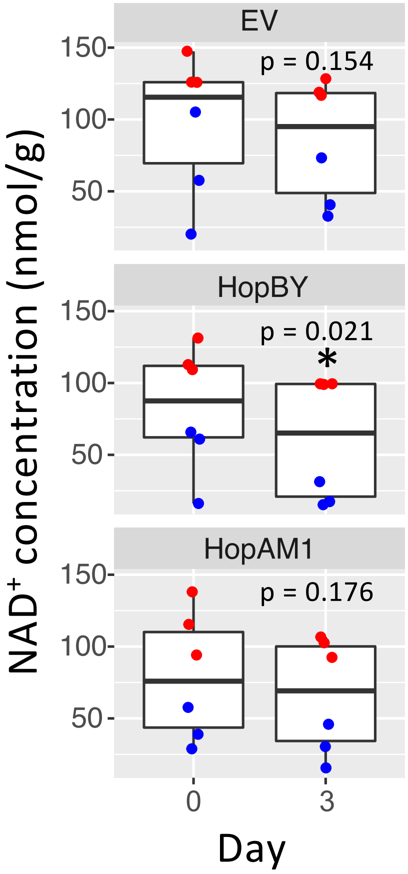

Figure S8: NAD$^+$ quantification in *A. thaliana* leaves inoculated with D36E expressing HopBY, HopAM1 or carrying the empty vector at day 0 and 3 post inoculation. NAD$^+$ was quantified using LCMS using a concentration curve of NAD$^+$. * denotes statistical significance using a t-test ($p<0.05$). The p value for each treatment is presented. The analysis is based on results of two independent experiments with data points colored in red and blue respectively.

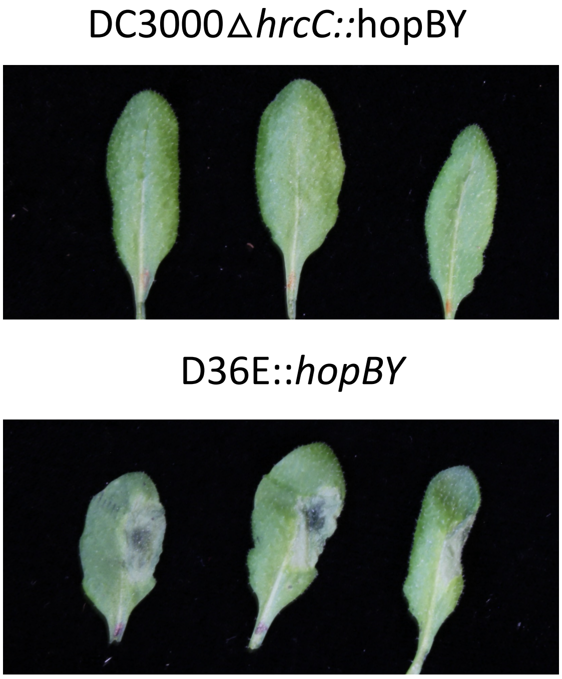

Figure S9. HopBY symptoms are dependent on a functional T3 secretion system
Representative images of *A. thaliana* leaves inoculated with either DC3000Δ*hrcC::*hopBY or D36E::*hopBY* at a concentration of 1 x 10^8 CFU/mL and imaged 24 hours post inoculation.

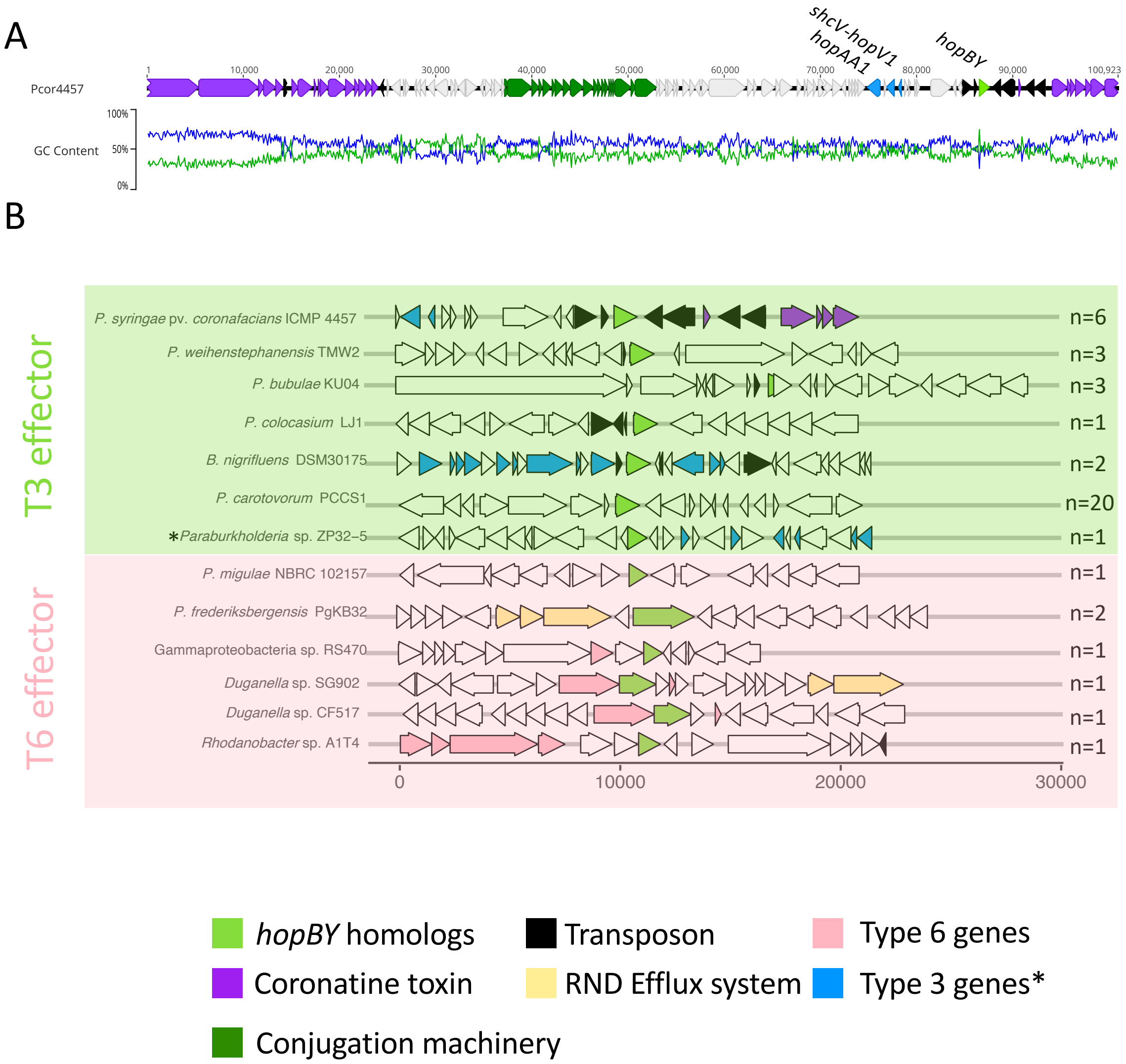

Figure S10. Genomic location of the *hopBY* homologs.

A. Genome region map of *hopBY* in ParICMP4457_Contig_64. Open reading frames are colour-coded to indicate functions. GC content is shown with GC (blue) and AT (green) percentage determined over a sliding window of 48 nucleotides.

B. Genome regions containing *hopBY* homologs in various bacteria. Gene content within a 30 Kb region surrounding the *hopBY* homolog (in green) is presented for each genome. The number of genomes containing the same gene layout is also shown. Open reading frames are color-coded to indicate functions. Genomes containing *hopBY* homologs as T3E or T6Es are labelled. The asterisk labels *Paraburkholderia* sp. Zp32-5, which encodes a *hopBY* homolog predicted to be a T3E although this bacterium has the Hrp2 T3S. All other bacteria that encode *hopBY* homologs as T3E have the Hrp1 T3S.

Table S1 PFAM accessions for each enzyme group used to construct HMM

PFAM	Group	Description	Seed alignment size
**PF01582**	TIR	Toll-Interleukin receptor	23
**PF08937**	TIR	MTH538 TIR-like domain	27
**PF10137**	TIR	Predicted nucleotide-binding protein containing TIR-like domain	49
**PF13676**	TIR	TIR domain	458
**PF18567**	TIR	Toll-Interleukin receptor	26
**PF02267**	ADPR cyclase	Rib_hydrolayse	27
**PF01129**	ART	NAD(P)+-protein-arginine ADP-ribosyltransferase	8
**PF01375**	ART	Heat-labile enterotoxin family	6
**PF01885**	ART	RNA 2'-phosphotransferase, Tpt1 / KptA family	222
**PF02027**	ART	RolB/RolC glucosidase family	13
**PF00644**	ART	Poly(ADP-ribose) polymerase catalytic domain	27
**PF02917**	ART	Pertussis toxin	2
**PF03496**	ART	ADP-ribosyltransferase exoenzyme	21
**PF06108**	ART	Protein of unknown function (DUF952)	188
**PF08808**	ART	RES domain	180
**PF09009**	ART	Exotoxin A catalytic	2
**PF09143**	ART	AvrPphF-ORF-2	4
**PF09475**	ART	Dot/Icm secretion system protein	3
**PF10386**	ART	Protein of unknown function (DUF2441)	4
**PF12120**	ART	Rifampin ADP-ribosyl transferase	32
**PF13151**	ART	Protein of unknown function (DUF3990)	86
**PF14021**	ART	Tuberculosis necrotizing toxin	100
**PF14487**	ART	ssDNA thymidine ADP-ribosyltransferase, DarT	47
**PF15633**	ART	HYD1 signature containing ADP-ribosyltransferase	6
**PF18648**	ART	Tse2 ADP-ribosyltransferase toxins	5
**PF18760**	ART	ADP-Ribosyltransferase in polyvalent proteins	15
**PF19048**	ART	SidE mono-ADP-ribosyltransferase domain	3
**PF00293**	NUDIX	NUDIX domain	127
**PF03559**	NUDIX	NDP-hexose 2,3-dehydratase	70
**PF09296**	NUDIX	NADH pyrophosphatase-like rudimentary NUDIX domain	36
**PF13869**	NUDIX	Nucleotide hydrolase	17
**PF14443**	NUDIX	DBC1	23
**PF14815**	NUDIX	NUDIX domain	384
**PF15916**	NUDIX	Domain of unknown function (DUF4743)	30
**PF16705**	NUDIX	NUDIX, or N-terminal NPxY motif-rich, region of KRIT	7

**PF01156**	Nucleoside N-ribohydrolase	Inosine-uridine preferring nucleoside hydrolase	664
**PF02146**	Sirtuin	Sir2 family	18
**PF00149**	PDE	Calcineurin-like phosphoesterase	324
**PF00233**	PDE	3'5'-cyclic nucleotide phosphodiesterase	247
**PF01663**	PDE	Type I phosphodiesterase / nucleotide pyrophosphatase	67

Table S2 Number of orthogroups with NADase domains identified in *P. syringae*

	HHsearch	HHsearch unique	HMM	HMM unique	Shared	Complete	Core (>95% genomes)	Accessory
TIR	65	33	34	2	32	**67**	5	62
NUDIX	109	81	30	2	28	**111**	38	73
ART	140	112	34	6	28	**146**	28	118
PDE	226	186	40	0	40	**226**	53	173
NH	8	3	5	0	5	**8**	2	6
SIR	15	12	3	0	3	**15**	2	13
ADPRC	1	1	0	0	0	**1**	0	1
Total	564	428	146	10	136	**574**	128	446

Table S3 Bacterial strains and constructs used in this study

Strain/construct	Purpose	Reference
P. syringae pv. tomato DC3000	Expression, secretion assay, rifR	(38)
Pst DC3000 Δ*hrcC*	Expression, secretion assay, rifR	(1)
Pst DC3000 D36E	Virulence assays, rifR	(2)
P. coronafaciens NCPPB 2397	Source of gDNA for cloning effector	(39)
GV3101	Agroinfiltration, gmR, rifR	(40)
GV3101:P19	*Agrobacterium tumefaciens* with silencing suppressor for agroinfiltration	(41)
DH10β/StellarTM competant cells	Cloning	ThermoFisher, Takara
pucp20TK	Broad host-range vector for *Pseudomonas* expression, kmR	(42)
pucp20TK:OG18056-HA	Expression of effector in *Pseudomonas*, kmR	This study
pucp20TK:OG18056-E305A-HA	Expression of effector in *Pseudomonas*, kmR	This study
pucp20TK:hopAM1-HA	Expression of effector in *Pseudomonas*, kmR	This study
pucp20TK:hopAM1-E191A-HA	Expression of effector in *Pseudomonas*, kmR	This study
pBBR1MCS-5	Broad host range vector for Pseudomonas expression, gmR	(43)
pBBR1MCS-5: avrRps4	Expression of effector in *Pseudomonas*, gmR	(5)
pICH51266	35s promoter, spR	(3) (addgene 50267)
pICSL01005	Level 1 acceptor CDS no stop codon, carbR	(3) (addgene 174575)
pICSL50007	3xFlag (C-terminal), spR	(3) (addgene 50308)
pICH41414	35s terminator, spR	(3) (addgene 50337)
pICH47732	Level 0 acceptor, spR	(3) (addgene 48000)
pICSL01005::35s-OG18056-3xFLAG	Transient expression of effector in plants, carbR	This study
pICSL01005::35s-OG18056-E305A-3xFLAG	Transient expression of effector in plants, carbR	This study
StrepTag Ab-TIR histag in pET30a+	AbTIR expression in *E.coli*	(10)

Table S4 Primers used in this study

Primer	Purpose	Experiment	Sequence (5'-3')	Amplicon size (bp)
hopAM1_cdsF	Add infusion overhangs to synthesised gene product with HA tag	Bacterial expression	GGAGTTCAAGATGCACGCAAATCCT	888
hopAM1_cdsR			TATGACCATGATTACGAATTCTAAG	
hopAM1_promF	Amplify promoter with infusion overhangs	Bacterial expression	CAGGTCGACTCTAGAGGATCCAGGCAAAGTAGATGCCGCGT	246
hopAM1_promR			TAAAGGATTTGCGTGCATGTATGCCTCCAGACGTT	
OG18056_F	Amplify promoter and CDS with infusion overhangs and add HA tag	Bacterial expression	CGACTCTAGAGGATCTTTGTCTGCACCCTCTTAGGAAAAA	1359
OG18056_HA_R			CCATGATTACGAATTCTAAGCGTAATCTGGAACATCGTATGGGT ACGTCAGGTTGCCACGAT	
OG18056_R1 E305A	Site-directed mutagenesis of OG18056	Bacterial expression	GGATGGGCCGCGCCTTGGTGTTCCATGT	1176 (with OG18056_F)
OG18056_F2 E305A	Site-directed mutagenesis of OG18056	Bacterial expression	AGGCGCGGCCCATCCTCGACAGAAACAA	185 (with OG18056_HA_R)

Table S5 Protein accession numbers for Hrp1 T3S identification

Strain	Accession	Description
Pst	AAO54900	membrane-bound lytic murein transglycosylase D
Pst	AAO54901	type III transcriptional regulator HrpR
Pst	AAO54902	type III transcriptional regulator HrpS
Pst	AAO54903	type III helper protein HrpA1
Pst	AAO54904	type III helper protein HrpZ1
Pst	AAO54905	type III secretion protein HrpB
Pst	AAO54906	type III secretion protein HrcJ
Pst	AAO54907	type III secretion protein HrpD
Pst	AAO54908	type III secretion protein HrpE
Pst	AAO54909	type III secretion protein HrpF
Pst	AAO54910	type III secretion protein HrpG
Pst	AAO54911	type III secretion protein HrpT
Pst	AAO54912	negative regulator of hrp expression HrpV
Pst	AAO54913	RNA polymerase sigma factor HrpL
Pst	AAO54926	type III helper protein HrpK1
Pst	AAO54927	HrpK
Pss	AAY36238.1	HrpL
Pss	AAY36244.1	HrpJ
Pss	AAY36245.1	HrcV
Pss	AAY36246.1	HrcN
Pss	AAY36247.1	HrpV
Pss	AAY36248.1	HrpT
Pss	AAY36249.1	HrpG
Pss	AAY36250.1	HrpF
Pss	AAY36251.1	HrpE
Pss	AAY36252.1	HrpD
Pss	AAY36253.1	HrpB
Pss	AAY36254.1	HrpA
Pss	AAY36255.1	HrpS
Pss	AAY36256.1	HrpR
Pss	AAY36271.1	membrane-bound lytic murein transglycosylase-like protein
Pss	AAY36272.1	type III helper protein HrpW1
P.vir	AAT96136.1	type III transcriptional regulator HrpR
P.vir	AAT96137.1	type III transcriptional regulator HrpS
P.vir	AAT96138.1	type III helper protein HrpA2
P.vir	AAT96139.1	type III helper protein HrpZ1
P.vir	AAT96141.1	type III secretion protein HrpB
P.vir	AAT96150.1	type III secretion apparatus lipoprotein, YscJ/HrcJ family
P.vir	AAT96151.1	type III secretion protein HrpD
P.vir	AAT96153.1	type III secretion protein HrpE
P.vir	AAT96154.1	type III secretion protein HrpF

P.vir	AAT96155.1	type III secretion protein HrpG
P.vir	AAT96156.1	outer-membrane type III secretion protein HrcC
P.vir	AAT96158.1	type III secretion protein HrpT
P.vir	AAT96160.1	negative regulator of hrp expression HrpV
P.vir	AAT96161.1	Sigma-70 region 2:Sigma-70 region 4
P.vir	AAT96162.1	type III helper protein HrpK1
P.vir	AAT96163.1	outer-membrane type III secretion protein HrcC

Dataset S1. Metainformation for orthogroups identified as putative NADases in the *P. syringae* pangenome (see excel)

Dataset S2. NADase effectors identified using EffectiveT3 from the *P. syringae* pangenome (see excel)

Dataset S3. OG18056 homologues across bacteria are type III or type VI effectors (see excel)

Supplementary references

1. J. Yuan, S. Y. He, The *Pseudomonas syringae* Hrp Regulation and Secretion System Controls the Production and Secretion of Multiple Extracellular Proteins. *J Bacteriol* **178**, 6399-6402 (1996).
2. H. L. Wei *et al.*, *Pseudomonas syringae* pv. *tomato* DC3000 Type III Secretion Effector Polymutants Reveal an Interplay between HopAD1 and AvrPtoB. *Cell Host Microbe* **17**, 752-762 (2015).
3. C. Engler, R. Kandzia, S. Marillonnet, A one pot, one step, precision cloning method with high throughput capability. *PLoS One* **3**, e3647 (2008).
4. K. H. Choi, A. Kumar, H. P. Schweizer, A 10-min method for preparation of highly electrocompetent *Pseudomonas aeruginosa* cells: application for DNA fragment transfer between chromosomes and plasmid transformation. *J Microbiol Methods* **64**, 391-397 (2006).
5. K. H. Sohn, Y. Zhang, J. D. Jones, The Pseudomonas syringae effector protein, AvrRPS4, requires in planta processing and the KRVY domain to function. *Plant J* **57**, 1079-1091 (2009).
6. U. K. Laemmli, Cleavage of structural proteins during the assembly of the head of bacteriophage T4. *Nature* **227**, 680-685 (1970).
7. H. Zhou, R. M. Morgan, S. Guttman, W. Ma, Allelic Variants of the *Pseudomonas syringae* Type III Effector HopZ1 Are Differentially Recognized by Plant Resistance Systems. *MPMI* **22**, 176-189 (2009).
8. B. A. Vinatzer, J. Jelenska, J. T. Greenberg, Bioinformatics Correctly Identifies Many Type III Secretion Substrates in the Plant Pathogen *Pseudomonas syringae* and the Biocontrol Isolate *P. fluorescens* SBW25. *MPMI* **18**, 877-888 (2005).
9. Z. Duxbury *et al.*, Induced proximity of a TIR signaling domain on a plant-mammalian NLR chimera activates defense in plants. *Proc Natl Acad Sci U S A* **117**, 18832-18839 (2020).
10. K. Essuman *et al.*, TIR Domain Proteins Are an Ancient Family of NAD(+)-Consuming Enzymes. *Curr Biol* **28**, 421-430 e424 (2018).
11. M. S. Drenichev *et al.*, A role for 3'-O-beta-D-ribofuranosyladenosine in altering plant immunity. *Phytochemistry* **157**, 128-134 (2019).
12. C. Jain, L. Rodriguez-R, A. M. Phillippy, K. T. Konstantinidis, S. Aluru, High throughput ANI analysis of 90K prokaryotic genomes reveals clear species boundaries. *Nat Commun* **9**, 5114 (2018).
13. A. Levy *et al.*, Genomic features of bacterial adaptation to plants. *Nat Genet* **50**, 138-150 (2018).
14. A. Gurevich, V. Saveliev, N. Vyahhi, G. Tesler, QUAST: quality assessment tool for genome assemblies. *Bioinformatics* **29**, 1072-1075 (2013).

15. D. H. Parks, M. Imelfort, C. T. Skennerton, P. Hugenholtz, G. W. Tyson, CheckM: assessing the quality of microbial genomes recovered from isolates, single cells, and metagenomes. *Genome Res* **25**, 1043-1055 (2015).
16. T. Seemann, Prokka: rapid prokaryotic genome annotation. *Bioinformatics* **30**, 2068-2069 (2014).
17. D. M. Emms, S. Kelly, OrthoFinder: phylogenetic orthology inference for comparative genomics. *Genome Biol* **20**, 238 (2019).
18. J. Thompson, D. Higgins, T. Gibson, CLUSTAL W: improving the sensitivity of progressive multiple sequence alignment through sequence weighting, position-specific gap penalties and weight matrix choice. *Nucleic Acids Res* **22**, 4673-4680 (1994).
19. J. Castresana, Selection of Conserved Blocks from Multiple Alignments for Their Use in Phylogenetic Analysis. *Mol Biol Evol* **17**, 540–552 (2000).
20. B. Q. Minh *et al.*, IQ-TREE 2: New Models and Efficient Methods for Phylogenetic Inference in the Genomic Era. *Mol Biol Evol* **37**, 1530-1534 (2020).
21. J. Pei, B. H. Kim, N. V. Grishin, PROMALS3D: a tool for multiple protein sequence and structure alignments. *Nucleic Acids Res* **36**, 2295-2300 (2008).
22. J. Li *et al.*, SecReT6: a web-based resource for type VI secretion systems found in bacteria. *Environ Microbiol* **17**, 2196-2202 (2015).
23. M. Mirdita *et al.*, ColabFold - Making protein folding accessible to all. *BioRxiv* 10.1101/2021.08.15.456425 (2022).
24. S. B. Pandit, J. Skolnick, Fr-TM-align: a new protein structural alignment method based on fragment alignments and the TM-score. *BMC Bioinformatics* **9**, 531 (2008).
25. J. Eberhardt, D. Santos-Martins, A. F. Tillack, S. Forli, AutoDock Vina 1.2.0: New Docking Methods, Expanded Force Field, and Python Bindings. *J Chem Inf Model* **61**, 3891-3898 (2021).
26. R. Krivak, D. Hoksza, P2Rank: machine learning based tool for rapid and accurate prediction of ligand binding sites from protein structure. *J Cheminform* **10**, 39 (2018).
27. J. J. Irwin *et al.*, ZINC20-A Free Ultralarge-Scale Chemical Database for Ligand Discovery. *J Chem Inf Model* **60**, 6065-6073 (2020).
28. W. P. Feinstein, M. Brylinski, Calculating an optimal box size for ligand docking and virtual screening against experimental and predicted binding pockets. *J Cheminform* **7**, 18 (2015).
29. R.Core.Team, *R: A language and environment for statistical computing.* (R Foundation for Statistical Computing,, Vienna, Austria, 2020).
30. E. Paradis, K. Schliep, ape 5.0: an environment for modern phylogenetics and evolutionary analyses in R. *Bioinformatics* **35**, 526-528 (2019).
31. K. P. Schliep, phangorn: phylogenetic analysis in R. *Bioinformatics* **27**, 592-593 (2011).
32. D. Wilkins (2020) gggenes: Draw Gene Arrow Maps in 'ggplot2'. (R package version 0.4.1.).
33. H. Wickham, *ggplot2: Elegant Graphics for Data Analysis* (Springer-Verlag New York, 2016).
34. Y. Guangchuang, Using ggtree to Visualize Data on Tree-Like Structures. *Current Protocols in Bioinformatics* **69**, e96 (2020).
35. H. Wickham, Reshaping Data with the reshape Package. *Journal of Statistical Software* **21**, 1-20 (2007).
36. R. V. Lenth (2022) emmeans: Estimated Marginal Means, aka Least-Squares Means. R package version 1.7.3.

37. F. de Mendiburu, M. Yaseen (2020) agricolae: Statistical Procedures for Agricultural Research.R package version 1.4.0.
38. D. A. Cuppels, Generation and Characterization of Tn5 Insertion Mutations in *Pseudomonas syringae* pv. *tomato*. *Appl Environ Microbiol* **51**, 323-327 (1986).
39. C. S. Reddy, J. Godkin, A bacterial disease of bromegrass. *Phytopathology* **13**, 75-86 (1923).
40. M. Holsters *et al.*, The functional organization of the nopaline *A. tumefaciens* plasmid pTiC58. *Plasmid* **3**, 212-230 (1980).
41. D. C. Baulcombe, A. Molnar, Crystal structure of p19--a universal suppressor of RNA silencing. *Trends Biochem Sci* **29**, 279-281 (2004).
42. H. Zhou, R. Morgan, D. Guttman, W. Ma, Allelic Variants of the Pseudomonas syringae Type III Effector HopZ1 Are Differentially Recognized by Plant Resistance Systems. *Mol Plant Microbe Interact* **22**, 176-189 (2009).
43. M. Kovach *et al.*, Four new derivatives of the broad-host-range cloning vector pBBR1MCS, carrying different antibiotic-resistance cassettes. *Gene* **166**, 175-176 (1995).
